# Granisetron transdermal delivery system versus palonosetron in the prevention of long-delayed nausea and vomiting: a phase III randomized trial

**DOI:** 10.1093/oncolo/oyag007

**Published:** 2026-01-11

**Authors:** Xiaojun Liu, Yanchun Meng, Yiqun Du, Yuxin Mu, Gang Li, Hengyu Li, Xiaoxiang Guan, Jian Zhang

**Affiliations:** Department of Medical Oncology, Fudan University Shanghai Cancer Center, Shanghai 200032, China; Phase I Clinical Trial Center, Fudan University Shanghai Cancer Center, Shanghai 200032, China; Department of Oncology, Shanghai Medical College, Fudan University, Shanghai 200032, China; Department of Medical Oncology, Fudan University Shanghai Cancer Center, Shanghai 200032, China; Phase I Clinical Trial Center, Fudan University Shanghai Cancer Center, Shanghai 200032, China; Department of Oncology, Shanghai Medical College, Fudan University, Shanghai 200032, China; Department of Medical Oncology, Fudan University Shanghai Cancer Center, Shanghai 200032, China; Phase I Clinical Trial Center, Fudan University Shanghai Cancer Center, Shanghai 200032, China; Department of Oncology, Shanghai Medical College, Fudan University, Shanghai 200032, China; Department of Medical Oncology, Fudan University Shanghai Cancer Center, Shanghai 200032, China; Phase I Clinical Trial Center, Fudan University Shanghai Cancer Center, Shanghai 200032, China; Department of Oncology, Shanghai Medical College, Fudan University, Shanghai 200032, China; Minhang Branch, Fudan University Shanghai Cancer Center, Shanghai 201100, China; Department of Breast and Thyroid Surgery, Changhai Hospital, Naval Military Medical University, Shanghai 200433, China; Department of Oncology, The First Affiliated Hospital of Nanjing Medical University, Nanjing 210000, China; Department of Medical Oncology, Fudan University Shanghai Cancer Center, Shanghai 200032, China; Phase I Clinical Trial Center, Fudan University Shanghai Cancer Center, Shanghai 200032, China; Department of Oncology, Shanghai Medical College, Fudan University, Shanghai 200032, China

**Keywords:** granisetron transdermal delivery system, chemotherapy-induced nausea and vomiting, aprepitant, HEC, MEC

## Abstract

**Background:**

Chemotherapy-induced nausea and vomiting (CINV) significantly impact the patients’ quality of life. Whether the granisetron transdermal delivery system (GTDS) offers better protection than palonosetron against long-delayed (120-168 hours) CINV after highly or moderately emetogenic chemotherapy (HEC/MEC) had not been prospectively tested.

**Methods:**

A multicenter, randomized clinical study was conducted in China. Patients scheduled to receive either HEC or MEC were randomly assigned (1:1) to GTDS or palonosetron, each in combination with a neurokinin-1 receptor antagonist (NK1-RA) and dexamethasone. The primary endpoint was the complete response (CR; no vomiting and no rescue medication) rate during the long-delayed phase (120-168 hours), stratified by HEC and MEC categories, to demonstrate the superiority of GTDS over palonosetron.

**Results:**

Overall, 150 patients received either GTDS or palonosetron respectively. We found that the GTDS group demonstrated a significantly higher long-delayed CR rate (97.3%) than the palonosetron group (92%) (*P *= .04). This advantage was driven predominantly by the HEC subgroup (GTDS 97.5% vs palonosetron 90.8%, *P *= .028). No significant differences were observed between the groups for the acute (0-24 hours, 92.7% vs 90.0%; *P* = .412), delayed (24-120 hours, 80.0% vs 76.0%; *P* = .403), extended-delayed (24-168 hours, 80.7% vs 75.3%; *P* = .265), or overall (0-168 hours, 78.0% vs 74.0%; *P* = .417) phases.

**Conclusion:**

A GTDS-based triple antiemetic regimen can effectively control CINV associated with HEC or MEC. It provides a convenient alternative route for delivering granisetron for up to 7 days, with superior efficacy in controlling long-delayed CINV.

**Trial registration:**

Clinicaltrials.gov Identifier: NCT04912271 (in-house ethic number: YBCSG-21-04)

Lessons Learned There are some limitations in this study. First, double-blind studies are recommended to further confirm the value of GTDS in managing chemotherapy-induced nausea and vomiting. Second, the quadruple antiemetic regimen, including olanzapine, should be studied in future clinical trials given olanzapine’s notable efficacy in alleviating nausea. Third, future studies focus on specific populations (such as patients receiving only HEC) were needed to validate our results.

## Introduction

Chemotherapy-induced nausea and vomiting (CINV) is one of the most common side effects of chemotherapy in cancer patients, seriously affecting their quality of life and compliance.^1^ Mechanically, CINV involves intricate interactions between various neurotransmitters in the central nervous system and the afferent vagal nerve endings in the gastrointestinal tract.^2^^,^^3^ International guidelines recommend the prophylactic use of antiemetic drugs based on patients receiving highly emetogenic chemotherapy (HEC) or moderately emetogenic chemotherapy (MEC) regimens. Currently, the standard management of CINV mainly consists of 5-hydroxytryptamine receptor antagonists (5-HT3RAs), NK1 receptor antagonists and glucocorticoids, combined with olanzapine or not.^4–6^ Recent studies have shown that the addition of olanzapine to standard antiemetic regimens significantly improves the control of both acute and delayed CINV, with a notable reduction in the incidence of delayed nausea. However, the role of olanzapine in combination with GTDS has not been fully explored.

Despite the effectiveness of these regimens, a notable proportion of patients undergoing moderately or highly emetogenic chemotherapy still experience acute and delayed CINV. This treatment gap has spurred the development and introduction of newer agents aimed at enhancing control rates for these challenging symptoms. For 5-HT3RAs, the use of intravenous formulations can improve compliance compared to oral formulations, as intravenous drugs are only given on the day of chemotherapy. Nonetheless, intravenous 5-HT3RAs may cause infusion site reactions. To expand the treatment options for preventing CINV, new agents are needed. GTDS is capable of continuously administering granisetron via the skin, dispensing 3.3 mg of granisetron every 24 hours for up to 168 hours.^7^^,^^8^ GTDS offering a convenient non-invasive method for sustained antiemetic administration, has demonstrated non-inferior efficacy in controlling CINV when compared to other generation 5-HT3RAs.^9–12^

Depending on the timing of nausea and vomiting, CINV is generally divided into the acute phase (within 24 hours post-chemotherapy administration), the delayed phase (24-120 hours after chemotherapy initiation), the extended delayed phase (24-168 hours after chemotherapy), and the long-delayed phase (120-168 hours after chemotherapy). Despite the progress in antiemetic treatment making CINV manageable in the acute phase, controlling delayed phase and long-delayed phase (120-168 hours) continues to be challenging. While many clinical studies have evaluated CINV incidents within 120 hours, delayed phase of CINV frequently happens beyond this timeframe. Recent research found that nausea and vomiting even continued for over 120 hours after the start of chemotherapy treatment.^13^^,^^14^ A large clinical trial in Japan showed that 15%-25% of cancer patients treated with HEC or MEC experienced CINV at 120 and 168 hours.^15^ Chow et al. showed that long-delayed CINV has a similar severity to delayed CINV and affects many patients. There were 49% and 31% of patients receiving HEC suffering from delayed and long-delayed nausea, while 14% and 6% suffering from delayed and long-delayed vomiting, respectively.^16^ Recently, several studies have reported predictors of long-delayed CINV and symptoms in patients receiving HEC.^13^^,^^17^

According to current literature reports, studies on CINV in the long-delayed phase are indeed relatively scarce. To our knowledge, only a few studies have specifically explored the management of long-delayed CINV. For example, one study evaluated the efficacy of fosnetupitant versus fosaprepitant for preventing highly emetogenic CINV.^13^ The results showed that the CR rate reached 86.5% vs 81.4% in the long-delayed phase. These studies indicate that the management of long-delayed CINV is still an area that requires further exploration.

Our study aimed to address this critical gap by thoroughly evaluating the efficacy and tolerability of GTDS in comparison to palonosetron. This research sought to provide valuable insight into optimizing antiemetic strategies for cancer patients undergoing long-delayed CINV, with the goal of improving their overall treatment experience. Future research could consider further validating the effectiveness of relevant treatment regimens in non-transdermal delivery systems to better meet clinical needs.

## Methods

### Study design and treatment

Firstly, patients were randomly assigned to the GTDS or the palono setron group at a 1:1 ratio, stratified by whether they received highly emetogenic chemotherapy or moderately emetogenic chemotherapy. The study excluded patients who had received 5-HT3 receptor antagonists (5-HT3RAs), NK1 receptor antagonists, or any study drugs within 4 weeks before chemotherapy, to ensure that prior antiemetic exposure would not confound the study results. The length of each chemotherapy cycle was 14 or 21 days. This study evaluated the efficacy of GTDS or palonosetron plus NK-1 receptor antagonist and dexamethasone in patients receiving multiple cycles of chemotherapy, with a focus on the first cycle. We arranged the interventional treatment as follows. GTDS group (experimental group): Receive one transdermal patch of granisetron applied to the upper arm 24-48 hours before chemotherapy on Day 1 and kept in place for 7 days. Aprepitant, on the first day of chemotherapy, 1 hour before, 125 mg PO on Day 1, and 80 mg PO on Days 2-3. Dexamethasone, 30 minutes before chemotherapy on the first day, 12 mg, IV; 8 mg PO on Days 2-4. Palonosetron group (control group): Intravenous injection of a single dose of palonosetron 0.25 mg 30 minutes before the start of chemotherapy on Day 1. Aprepitant, on the first day of chemotherapy, 1 hour before, 125 mg PO on Day 1, and 80 mg PO on Days 2-3. Dexamethasone, 30 minutes before chemotherapy on the first day, 12 mg, IV; 8 mg PO on Days 2-4. Efficacy endpoints in the acute (0-24 hours), delayed (24-120 hours), extended delayed (24-168 hours), long-delayed delayed (120-168 hours), and overall (0-168 hours) phases were examined. Long-delayed nausea and vomiting were defined as any emetic episodes occurring between 120 and 168 hours after chemotherapy administration, regardless of the intensity or frequency.

### Participants

The research was conducted in 3 hospitals in China, constituting a multicenter, randomized, phase III clinical study. The intention-to-treat population to undergo emetogenic chemotherapy. A total of 300 patients were recruited into this study between August 2021 and December 2023. Inclusion criteria were listed as follows: (1) female or male aged ≥18 years; (2) pathologically confirmed solid tumors; (3) the physical status score ECOG ≤2; (4) life expectancy of ≥3 months; (5) patients scheduled to receive HEC/MEC chemotherapy, and the main emetic drugs will be used within a single day; included HEC regimens comprised AC (epirubicin + cyclophosphamide), TAC (docetaxel + doxorubicin + cyclophosphamide), carboplatin AUC ≥4, and cisplatin-containing regimens. MEC regimens included TC (docetaxel + cyclophosphamide) and FOLFOX (oxaliplatin + leucovorin + 5-FU). (Reference: NCI Guidelines). (6) Patients who had never used granisetron; (7) in accordance with the indication of chemotherapy and basic requirements; (8) be able to read, understand, and complete patient diaries independently.

The primary exclusion criteria included (1) contraindicated to 5-HT3RAs, NK-1 receptor antagonist or dexamethasone; (2) patients who had used 5-HT3RAs, NK1 receptor antagonist or any study drugs within 4 weeks before chemotherapy; (3) any nausea and vomiting (II grade or above) within 72 hours before the start of chemotherapy; (4) according to the judgment of the investigators, there are concomitant diseases (including but not limited to hypertension, severe diabetes, active infection, thyroid disease, etc.) that seriously endanger the safety of the patient or affect the completion of the study; (5) patients scheduled to receive radiotherapy of whole body, brain or upper abdomen; and (6) confirmed by craniocerebral CT or MRI, patients with brain tumor lesions or patients taking drugs to treat brain tumors or epileptic symptoms.

### Ethic approval

The study was conducted in accordance with the Declaration of Helsinki and the Ethical Guidelines for Clinical Studies. The approval was obtained from the Institutional Review Board of participating hospitals, and written informed consent was provided by all patients prior to enrollment. The trial was registered prospectively at ClinicalTrials.gov (NCT04912271).

### Efficacy end points

We defined the phases of CINV as follows: acute phase (0-24 hours), delayed phase (24-120 hours), extended delayed phase (24-168 hours), long-delayed phase (120-168 hours), and overall phase (0-168 hours). Data on nausea and vomiting were collected from patients self-reported diaries over a 7-day period, starting from the day of their initial chemotherapy. The primary efficacy endpoint focused on evaluating the proportion of patients attaining a complete response (CR) in the long-delayed (120-168 hours) phase of CINV. A CR was defined as the absence of emetic episodes and the non-utilization of rescue medication. Secondary endpoints included the daily and cumulative frequency of emetic episodes within the 0-24 hour, 24-120 hour, 24-168 hour, and 0-168 hour intervals. Additionally, the study examined the proportion of patients achieving complete control (CC), characterized by the absence of emetic episodes, no requirement for rescue medication, and no more than mild nausea, within intervals of 0-24 hours, 24-120 hours, 120-168 hours, 24-168 hours, and 0-168 hours, and patient global satisfaction with antiemetic therapy. Patients’ satisfaction scaling with antiemetic therapy was assessed using a visualized 10 cm bar to gauge the impact of nausea and emesis, with higher scores indicative of reduced symptomatology. Quality of life was assessed using the Functional Living Index–Emesis (FLIE) questionnaire, administered on Day 1 through DAY 8. Treatment compliance was monitored through patch-adherence logs in the GTDS arm and self-reported dosing compliance in the palonosetron arm.

### Study visits

In this study, we employed the WeChat platform for patient follow-up to efficiently collect CINV data. Patients enrolled by scanning a QR code, which created an electronic CINV diary on their phones. They received training on reporting symptoms accurately. A daily reminder system ensured data integrity, with the research team contacting patients if diaries were overdue. Data were collected via WeChat, managed securely, and analyzed by authorized personnel. Patients could communicate with the team anytime for support, enhancing engagement and real-time monitoring. This approach improved research efficiency, reduced dropouts, and leveraged WeChat’s user-friendliness to ensure high participation and data quality.

### Evaluation procedure

The evaluation procedures commenced with the screening of consenting patients for eligibility within a 7-day period prior to the initiation of the study. The screening encompassed a thorough assessment, including physical examination, measurement of vital signs and weight, laboratory tests (complete blood count [CBC] with differential, platelet counts, blood chemistry, and urine analysis), review of past medical history, examination of concomitant medications, and a history of nausea and vomiting. Throughout the study duration, participants maintained patient diaries to meticulously document emetic episodes, the use of rescue medication, patch adhesion, and the daily evaluation of nausea severity until day 4, as assessed by the National Cancer Institute Common Terminology Criteria for Adverse Events (NCI-CTCAE) version 5.0. These comprehensive evaluation procedures aimed to ensure a comprehensive understanding of the participants’ physical condition, treatment adherence, and safety profile throughout the study period.

### Statistical analysis

This study aims to evaluate the efficacy of granisetron transdermal delivery system (GTDS) and palonosetron groups. Because the release of GTDS could last up to 7 days, we hypothesized that GTDS may be more effective than palonosetron in preventing delayed CINV. The hypothesis of study posited that GTDS would exhibit superiority compared to palonosetron focusing on the patients with a long-delayed (120-168 hours) CR. The sample size of this study was calculated based on the following assumptions. Two previous studies^18^^,^^19^ showed that the CR rate of palonosetron during the long-delayed period was approximately 67%-91%. We estimated that the CR rate of the control group was about 88%. Considering that a 10.0% increase in this proportion would be a clinically significant effect size, we calculated that we would need to recruit 272 cases, with α = 0.05 and β = 0.1. We increased the sample size to 300 patients (150 per group) considering a dropout rate of 10%.

Efficacy analyses were assessed in the intention-to-treat group, including all the patients assigned at random. For the primary end point, chi-square or Fisher exact test was utilized for comparison of categorical variables, while *t* test was utilized for comparison of continuous variables. *P* < .05 was considered statistically significant. The entire statistical analysis was performed using IBM SPSS Statistics version 27.0. A retrospective investigative study was conducted to determine if the GTDS versus palonosetron impact varies across different covariate groups. This included variables like patient factors such as gender and age and the type of antiemetic prophylaxis used (ie, treatment group). This rigorous approach ensured the robustness and reliability of the study’s findings through appropriate hypothesis testing and statistical significance determination.

## Results

### Demographics and clincial characteristics

**Figure 1.**
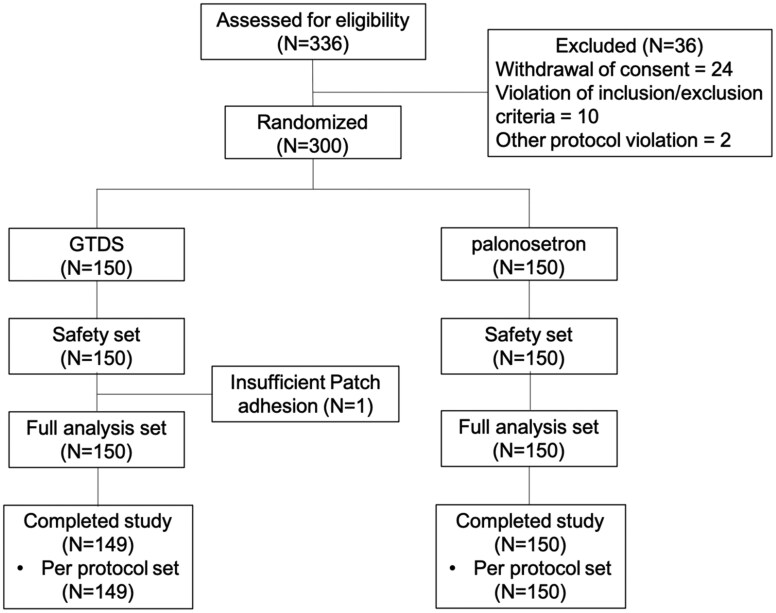
Study design of clinical trial. This diagram illustrates the flow of patients from screening to final analysis, including randomization into GTDS and palonosetron arms, stratified by chemotherapy emetogenicity (HEC vs. MEC).

A total of 336 patients assessed for eligibility, 300 patients from 3 medical institutions in China were recruited in this study between August 2021 and December 2023, including 240 patients receiving high emetic chemotherapy and 60 patients receiving moderate emetic chemotherapy. These patients were randomized into the GTDS group and the palonosetron group. One patient receiving high emetic chemotherapy did not complete GTDS intervention due to insufficient patch adhesion (Figure 1). The demographic and clinical information of patients was compared between groups and there were no significant differences in gender, age, body surface area, medical history (Table S1—see online supplementary material).

## Efficacy

### Primary efficacy analysis

This study achieved the predefined primary endpoint. The long-delayed CR rates of GTDS group and palonosetron group were 97.3% and 92.0% (*P* = .04), indicating that GTDS has superiority over palonosetron in controlling long-delayed CR (Table 1).

**Table 1.** Primary and secondary efficacy end points in all patients (intention-to-treat).

Endpoints	GTDS (*N* = 150) (%)	Palonosetron (*N* = 150) (%)		Treatment difference (95% CI) (%)	*P* value
**Primary end point**					
**CR in long-delayed phase (120-168 h)**	146 (97.3)	138 (92.0)	5.3 (0.3, 10.4)		.04
**Secondary end points**					
**CR in acute phase (0-24 h)**	139 (92.7)	135 (90.0)	2.7 (−3.7, 9.0)		.41
**CR in delayed phase (24-120 h)**	120 (80.0)	114 (76.0)	4.0 (−5.4, 13.4)		.40
**CR in extended delayed phase (24-168 h)**	121 (80.7)	113 (75.3)	5.4 (−4.0, 14.7)		.27
**CR in overall phase (0-168 h)**	117 (78.0)	111 (74.0)	4.0 (−5.7, 13.7)		.42
**CC in acute phase (0-24 h)**	137 (91.3)	133 (88.7)	2.6 (−4.1, 9.4)		.44
**CC in delayed phase (24-120 h)**	119 (79.3)	108 (72.0)	7.3 (−2.3, 17.0)		.14
**CC in long-delayed phase (120-168 h)**	143 (95.3)	134 (89.3)	6.0 (0.02, 11.9)		.05
**CC in extended delayed phase (24-168 h)**	118 (78.6)	106 (70.6)	8.0 (−1.8, 17.8)		.11
**CC in overall phase (0-168 h)**	115 (76.6)	106 (70.6)	6.0 (−3.9, 15.9)		.24

CR, complete response; CC, complete control.

**Table 2.** Primary and secondary efficacy end points in high emetic chemotherapy (intention-to-treat).

Endpoints	GTDS (*N* = 120) (%)	Palonosetro*n* (*N* = 120) (%)	Treatment difference (95% CI) (%)	
**Primary end point**				
**CR in long-delayed phase (120-168 h)**	117 (97.5)	109 (90.8)		6.7 (0.8, 12.5)
**Secondary end points**				
**CR in acute phase (0-24 h)**	110 (91.6)	106 (88.3)		3.3 (−4.2, 10.9)
**CR in delayed phase (24-120 h)**	94 (78.3)	89 (74.2)		4.1 (−6.5, 14.9)
**CR in extended delayed phase (24-168 h)**	94 (78.3)	88 (73.3)		5.0 (−5.8, 15.8)
**CR in overall phase (0-168 h)**	91 (75.8)	86 (71.7)		4.1 (−6.9, 15.2)
**CC in acute phase (0-24 h)**	108 (90.0)	105 (87.5)		2.5 (−5.4, 10.4)
**CC in delayed phase (24-120 h)**	93 (77.5)	85 (70.8)		6.7 (−4.3, 17.7)
**CC in long-delayed phase (120-168 h)**	115 (95.8)	107 (89.1)		6.7 (0.0, 13.2)
**CC in extended delayed phase (24-168 h)**	93 (77.5)	83 (69.2)		8.3 (−2.8, 19.4)
**CC in overall phase (0-168 h)**	90 (75.0)	83 (69.2)		5.8 (−5.4, 17.1)

CR, complete response; CC, complete control.

**Table 3.** Primary and secondary efficacy end points in moderate emetic chemotherapy (intention-to-treat).

Endpoints	GTDS (*N* = 30) (%)	Palonosetron (*N* = 30) (%)		Treatment difference (95% CI) (%)
**Primary end point**				
**CR in long-delayed phase (120-168 h)**	29 (96.7)	29 (96.7)	0 (−9.0, 9.0)	
**Secondary end points**				
**CR in acute phase (0-24 h)**	29 (96.7)	29 (96.7)	0 (−9.0, 9.0)	
**CR in delayed phase (24-120 h)**	26 (86.6)	25 (83.3)	3.3 (−14.7, 21.3)	
**CR in extended delayed phase (24-168 h)**	27 (90.0)	25 (83.3)	6.7 (−10.4, 23.7)	
**CR in overall phase (0-168 h)**	26 (86.6)	23 (76.6)	10 (−9.4, 29.4)	
**CC in acute phase (0-24 h)**	29 (96.6)	28 (93.3)	3.3 (−7.6, 14.3)	
**CC in delayed phase (24-120 h)**	26 (86.6)	23 (76.6)	10 (−9.4, 29.4)	
**CC in long-delayed phase (120-168 h)**	28 (93.3)	27 (90.0)	3.3 (−10.6, 17.2)	
**CC in extended delayed phase (24-168 h)**	25 (83.3)	23 (76.6)	6.7 (−13.5, 26.8)	
**CC in overall phase (0-168 h)**	25 (83.3)	23 (76.6)	6.7 (−13.5, 26.8)	

CR, complete response; CC, complete control.

### Secondary efficacy analyses

**Figure 2.**
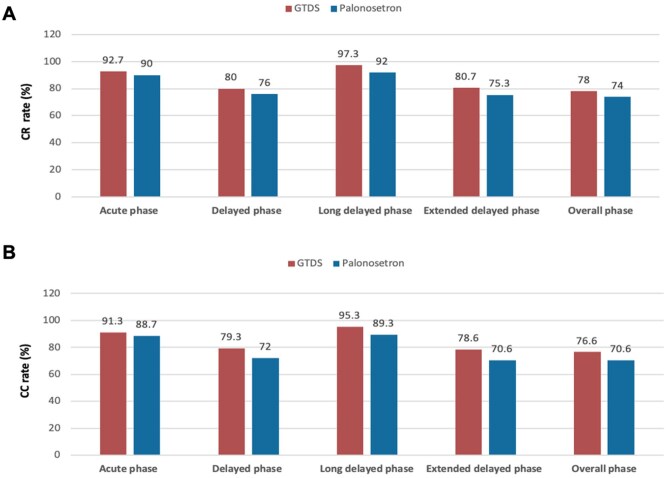
Complete response (CR) and complete control (CC) rates across time intervals in the overall population. (A) The CR (no vomiting and no rescue medication) rates during the acute (0-24 hours), delayed (24-120 hours), long-delayed (120-168 hours), extended delayed (24-168 hours), and overall (0-168 hours) phases in the cohort of all patients were calculated. (B) The CC (no emetic episodes, no rescue medication, and no more than mild nausea) rates during the acute (0-24 hours), delayed (24-120 hours), long-delayed (120-168 hours), extended delayed (24-168 hours), and overall (0-168 hours) phases in the cohort of all patients were calculated.

Figure 2A shows the different phase rates of CR and CC. Overall, there was a higher CR and CC rate in GTDS compared with palonosetron, though there was no significant difference (*P* > .05). Notably, the CC rate of long-delayed phase was higher in GTDS group than control group (95.3% vs 89.3) (*P* = .051), indicating that GTDS had certain advantage in preventing long-delayed nausea (Figure 2B).

**Figure 3.**
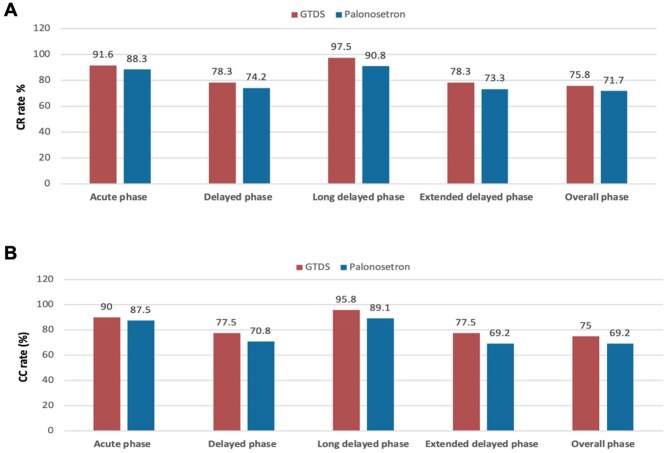
Complete response (CR) and complete control (CC) rates in the high emetogenic chemotherapy (HEC) subgroup. (A) The CR (no vomiting and no rescue medication) rates during the acute (0-24 hours), delayed (24-120 hours), long-delayed (120-168 hours), extended delayed (24-168 hours), and overall (0-168 hours) phases in the cohort of high emetic chemotherapy were calculated. (B) The CC (no emetic episodes, no rescue medication, and no more than mild nausea) rates during the acute (0-24 hours), delayed (24-120 hours), long-delayed (120-168 hours), extended delayed (24-168 hours), and overall (0-168 hours) phases in the cohort of high emetic chemotherapy were calculated.

**Figure 4.**
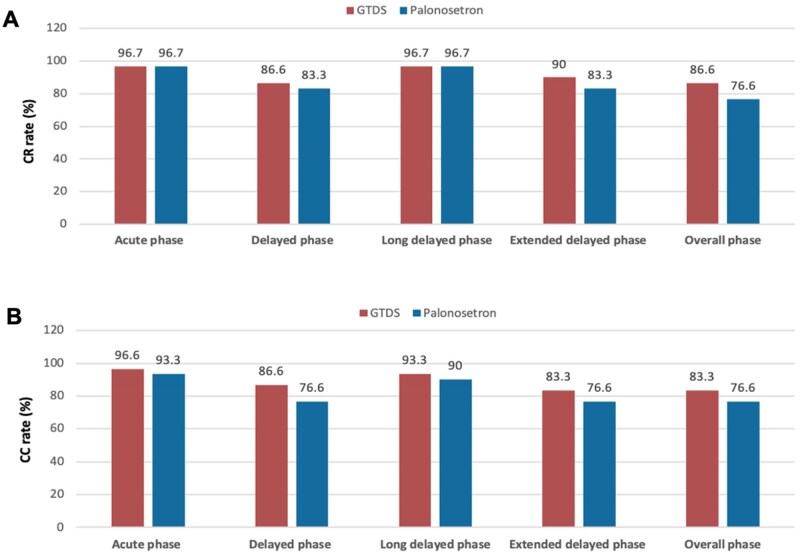
Complete response (CR) and complete control (CC) rates in the moderate emetogenic chemotherapy (MEC) subgroup. (A) The CR (no vomiting and no rescue medication) rates during the acute (0-24 hours), delayed (24-120 hours), long-delayed (120-168 hours), extended delayed (24-168 hours), and overall (0-168 hours) phases in the cohort of moderate emetic chemotherapy were calculated. (B) The CC (no emetic episodes, no rescue medication, and no more than mild nausea) rates during the acute (0-24 hours), delayed (24-120 hours), long-delayed (120-168 hours), extended delayed (24-168 hours), and overall (0-168 hours) phases in the cohort of moderate emetic chemotherapy were calculated.

Subgroup analyses were pre-specified to evaluate the efficacy of GTDS and palonosetron in patients receiving highly emetogenic chemotherapy (HEC) and moderately emetogenic chemotherapy (MEC). Examination of CR and CC in these subgroups showed no significant differences between the GTDS and palonosetron arms, except for the long-delayed CR rate in the HEC cohort (Figures 3 and 4). In the high emetic chemotherapy subgroup (*n* = 240), the GTDS group demonstrated a significantly higher long-delayed CR rate compared to palonosetron (97.5% vs 90.8%; *P* = .028) (Figure 3A, Table 2). Similarly, the long-delayed CC rate was numerically higher with GTDS (95.8% vs 89.1%; *P* = .05) (Figure 3B, Table 2).

**Figure 5.**
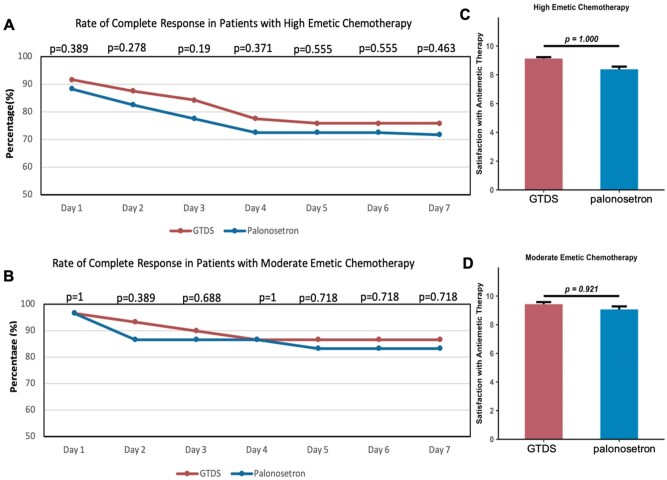
Daily complete response (CR) rates and patient satisfaction by chemotherapy subgroup. (A) Analysis of complete response rate in high emetic chemotherapy cohort on any day of follow-up. (B) Analysis of complete response rate in moderate emetic chemotherapy cohort on any day of follow-up. (C) Satisfaction with antiemetic therapy in high emetic chemotherapy cohort. (D) Satisfaction with antiemetic therapy in moderate emetic chemotherapy cohort. HEC, highly emetogenic chemotherapy; MEC, moderately emetogenic chemotherapy; OR, odds ratio.

In contrast, the cohort of moderate emetic chemotherapy (*n* = 60) showed no statistically significant differences between treatment arms for any endpoint (Figure 4, Table 3).The rates of CR after chemotherapy at 24-hour interval were also compared between groups in both cohorts (Figure 5A and B). The GTDS group showed higher control rate in CR compared with the palonosetron group. The subgroup analysis in the MEC cohort (*n* = 60) was underpowered to detect statistical significance due to the limited sample size. However, the interaction test suggested that both HEC and MEC populations may benefit from GTDS.

#### Safety

In the GTDS group, adverse events included gastrointestinal symptoms (nausea, vomiting, diarrhea, constipation) and systemic symptoms (fatigue, headache, dizziness). One patient had an issue with patch adhesion. Rescue medication used was ondansetron for Grade 3 nausea and vomiting. In the palonosetron group, adverse events were similar, with additional reports of insomnia and muscle pain. Rescue medication included ondansetron for Grade 3 nausea.

### Exploratory analysis

**Figure 6.**
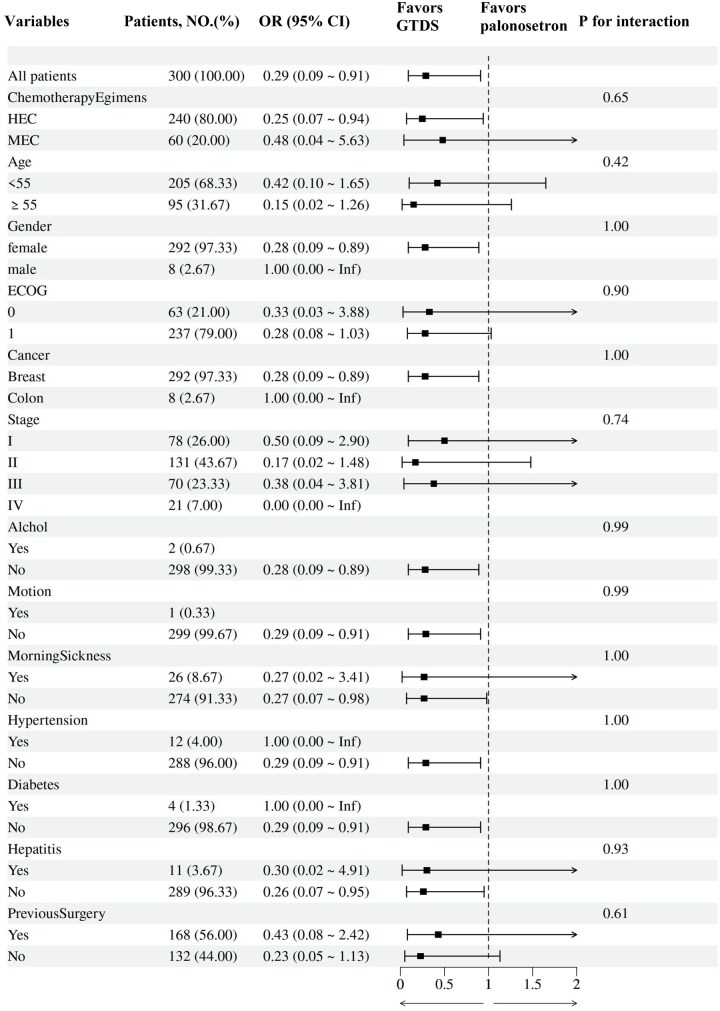
Forest plot of subgroup analysis for long-delayed complete response (CR) (120-168 h).

A retrospective exploratory analysis was conducted to explore the impact of the therapeutic approaches differed across distinct subgroups (Figure 6). The interaction analyses indicated that there was no interaction between subgroups no matter in the patient factors and the type of antiemetic prophylaxis.

### Satisfaction

Patient satisfaction with antiemetic therapy was assessed using a visual analog scale. The granisetron group demonstrated non-inferior satisfaction compared to the palonosetron group in both high-emetic and moderate-emetic chemotherapy cohorts (Figure 5C and D).

## Conclusions

GTDS offers a practical, non-invasive alternative for sustained antiemetic coverage, particularly beneficial in HEC settings. These findings support its integration into clinical practice to improve long-delayed CINV control.

## Discussion

Current antiemetic guidelines (eg, 2023 NCCN v2.2023, 2023 MASCC/ESMO) recommend triple therapy with a 5-HT_3_ receptor antagonist (5-HT_3_RA), a neurokinin-1 receptor antagonist (NK_1_-RA) and dexamethasone as standard prophylaxis for HEC and selected MEC.^4^^,^^6^^,^^20^ Palonosetron, as the second-generation 5HT3RAs, has demonstrated greater efficacy than the first generation in preventing acute and delayed nausea and vomiting induced by both highly and moderately emetogenic chemotherapy.^21^^,^^22^ Initially, we hypothesized that intravenous palonosetron would outperform the GTDS. However, our findings revealed that GTDS provided superior protection against long-delayed emesis (120-168 hours), particularly in patients receiving HEC, while maintaining a comparable safety profile.

Olanzapine, an atypical antipsychotic, has demonstrated significant efficacy in preventing both acute and delayed CINV when added to standard antiemetic regimens.^4–6^ A recent meta-analysis showed that the addition of olanzapine to standard antiemetic therapy significantly increased the CR rate in both the acute and delayed phases of CINV, with a relative risk reduction of 20%-30% for vomiting episodes. Additionally, olanzapine has been found to be particularly effective in managing delayed nausea, with studies reporting a 30%-40% reduction in the incidence of delayed nausea when compared to placebo.^23^ It is important to highlight that this study focused solely on evaluating the efficacy of the GTDS vs palonosetron within a triple antiemetic regimen, which did not include olanzapine. Future research could consider investigating the role of olanzapine in combination with GTDS or other antiemetic agents to further optimize the management of CINV.

According to the previous study, the evaluation intervals for CINV were limited to the first 120 hours after chemotherapy. Recently, an increasing number of studies have revealed that some patients experience nausea or vomiting beyond 120 hours.^15^^,^^24^^,^^25^ Research has shown that extending the use of aprepitant to 5 days can significantly reduce the incidence of long-delayed CINV.^24^ Hata et al. also confirmed that fosnetupitant and fosaprepitant have similar CR of long-delayed CINV in patients receiving HEC.^13^ However, the effect of GTDS in long-delayed phase remains unknown and needs more evidence.

The present randomized trial is the first to focus on the frequency of long-delayed nausea and vomiting among patients treated with GTDS and palonosetron following HEC and MEC. We demonstrate that patients receiving GTDS achievesd a 97.3% CR rate during this interval, exceeding palonosetron (92%; *P* = .040). The advantage is most pronounced in the HEC subgroup (97.5% vs 90.8%; *P* = .028), an effect plausibly attributable to the continuous transdermal input that maintains therapeutic granisetron levels for the entire 7-day period.^26^ The MEC subgroup analysis (*n* = 60) was underpowered to detect statistical significance due to limited sample size. Although, our interaction test suggested that both the population of HEC and MEC may benefit from GTDS.

Secondary efficacy endpoints—CR and CC rates during the acute (0-24 hours), delayed (24-120 hours), extended delayed (24-168 hours), and overall (0-168 hours) phases were generally higher in the GTDS arm than in the palonosetron arm, but the differences were not statistically significant. The fact that a relatively large number of patients experienced CINV beyond 120 hours underscores the need for extended evaluation of antiemetic medications. This finding suggests an unmet clinical need that has not been addressed in previous studies. The NK1 receptor antagonists, and dexamethasone, may therefore offer improved prophylaxis for this vulnerable period.

Safety and tolerability were comparable between the 2 arms. No clinically meaningful changes in laboratory parameters, vital signs, or electrocardiographic intervals were observed. The safety profile of GTDS was consistent with previous reports and with the known pharmacology of granisetron. Adverse events, most commonly constipation—were generally mild to moderate in intensity, and their incidence did not differ significantly between groups. Serious adverse events were rare and were judged to be related to the underlying malignancy or concomitant chemotherapy rather than to study medication. No clinically meaningful changes in laboratory parameters, vital signs, or electrocardiographic intervals were observed. The safety profile of GTDS was consistent with previous reports and with the known pharmacology of granisetron.

Overall, our study highlights the potential benefits of GTDS in managing long-delayed CINV, which is often overlooked in clinical practice. The results indicate that incorporating GTDS into antiemetic strategies may significantly improve the management of CINV, particularly for patients receiving highly emetogenic chemotherapy. However, there are several limitations to our study that should be acknowledged. First, the open-label design may introduce bias, double-blind studies are recommended to further confirm the value of GTDS in managing CINV. Second, future research should focus on optimizing the use of GTDS in combination with other antiemetic agents, such as olanzapine, to further enhance the efficacy of CINV management. Third, the predominantly breast cancer cohort with limited male representation may affect the generalizability of our findings. Future studies should focus on specific populations, such as patients with other types of cancer, male patients and patients receiving only HEC, are needed to validate our results. In clinical practice, the adoption of GTDS could provide an effective alternative for sustained antiemetic coverage, improving patient outcomes and overall treatment experience.

## Supplementary Material

oyag007_Supplementary_Data

## Data Availability

The data are available from the corresponding author on reasonable request.

## References

[1] Piechotta V , Adams A, Haque M, et al. Antiemetics for adults for prevention of nausea and vomiting caused by moderately or highly emetogenic chemotherapy: a network meta-analysis. Cochrane Database Syst Rev. 2021;11:CD012775. 10.1002/14651858 34784425 PMC8594936

[2] Navari RM. Management of chemotherapy-induced nausea and vomiting: focus on newer agents and new uses for older agents. Drugs. 2013;73:249-262. 10.1007/s40265-013-0019-1 23404093

[3] Yokokawa T , Suzuki K, Tsuji D, et al. Influence of menopause on chemotherapy-induced nausea and vomiting in highly emetogenic chemotherapy for breast cancer: a retrospective observational study. Cancer Med. 2023;12:18745-18754. 10.1002/cam4.6494 37676079 PMC10557896

[4] Hesketh PJ , Kris MG, Basch E, et al. Antiemetics: ASCO guideline update. J Clin Oncol. 2020;38:2782-2797. 10.1200/JCO.20.01296 32658626

[5] Herrstedt J , Clark-Snow R, Ruhlmann CH, et al. 2023 MASCC and ESMO guideline update for the prevention of chemotherapy- and radiotherapy-induced nausea and vomiting. ESMO Open. 2024;9:102195. 10.1016/j.esmoop.2023.102195 38458657 PMC10937211

[6] Kennedy SKF , Goodall S, Lee SF, et al. 2020 ASCO, 2023 NCCN, 2023 MASCC/ESMO, and 2019 CCO: a comparison of antiemetic guidelines for the treatment of chemotherapy-induced nausea and vomiting in cancer patients. Support Care Cancer. 2024;32:280. 10.1007/s00520-024-08462-x 38594320

[7] Midani D , Parkman HP. Granisetron transdermal system for treatment of symptoms of gastroparesis: a prescription registry study. J Neurogastroenterol Motil. 2016;22:650-655. 10.5056/jnm15203 27400689 PMC5056574

[8] Ramadon D , McCrudden MT, Courtenay AJ, et al. Enhancement strategies for transdermal drug delivery systems: current trends and applications. Drug Deliv Transl Res. 2022;12:758-791. 10.1007/s13346-021-00909-6 33474709 PMC7817074

[9] Seol YM , Kim HJ, Choi YJ, et al. Transdermal granisetron versus palonosetron for prevention of chemotherapy-induced nausea and vomiting following moderately emetogenic chemotherapy: a multicenter, randomized, open-label, cross-over, active-controlled, and phase IV study. Support Care Cancer. 2016;24:945-952. 10.1007/s00520-015-2865-8 26265119

[10] Yang Y , Zhang L. Granisetron transdermal delivery system is effective in the control of chemotherapy-induced nausea and vomiting in patients receiving moderately emetogenic chemotherapy (MEC) or highly emetogenic chemotherapy (HEC) in China. Chin Clin Oncol. 2017;6:14. 10.21037/cco.2017.04.02 28482667

[11] Sun S , Ko YH, Jin JY, et al. Efficacy of the granisetron transdermal system for the control of nausea and vomiting induced by highly emetogenic chemotherapy: a multicenter, randomized, controlled trial. Korean J Intern Med. 2023;38:406-416. 10.3904/kjim.2020.359 35263841 PMC10175860

[12] Armbruster SD , Fellman BM, Jhingran A, et al. A phase III study of transdermal granisetron versus oral ondansetron for women with gynecologic cancers receiving pelvic chemoradiation. Support Care Cancer. 2021;29:213-222. 10.1007/s00520-020-05484-z 32338316

[13] Hata A , Okamoto I, Inui N, et al. Randomized, double-blind, phase III study of fosnetupitant versus fosaprepitant for prevention of highly emetogenic chemotherapy-induced nausea and vomiting: CONSOLE. J Clin Oncol. 2022;40:180-188. 10.1200/JCO.21.01315 34793245 PMC8718175

[14] Hata A , Shiraishi Y, Inui N, et al. Exploratory analysis comparing fosnetupitant versus. fosaprepitant for prevention of highly emetogenic chemotherapy-induced nausea and vomiting (CINV): a randomized, double-blind, phase 3 study (CONSOLE). Oncol Ther. 2022;10:253-262. 10.1007/s40487-022-00188-2 35246827 PMC9098704

[15] Tamura K , Aiba K, Saeki T, et al. Testing the effectiveness of antiemetic guidelines: results of a prospective registry by the CINV Study Group of Japan. Int J Clin Oncol. 2015;20:855-865. 10.1007/s10147-015-0786-7 25681876

[16] Chow R , Yin LB, Baqri W, et al. Prevalence and predictors of long- delayed (> 120 h) chemotherapy-induced nausea and vomiting (CINV)-a systematic review and individual patient data meta- analysis. Support Care Cancer. 2023;31:505. 10.1007/s00520-023-07978-y 37535218

[17] Zelek L , Debourdeau P, Bourgeois H, et al. A pragmatic study evaluating NEPA versus aprepitant for prevention of chemo- therapy-induced nausea and vomiting in patients receiving moder- ately emetogenic chemotherapy. Oncologist. 2021;26:e1870-e1879. 10.1002/onco.13888 34216177 PMC8488783

[18] Isoda A , Saito R, Komatsu F, et al. Palonosetron, aprepitant, and dexamethasone for prevention of nausea and vomiting after high-dose melphalan in autologous transplantation for multiple myeloma: a phase II study. Int J Hematol. 2017;105:478-484. 10.1007/s12185-016-2152-6 27873176

[19] Rozzi A , Nardoni C, Corona M, et al. Palonosetron for the prevention of chemotherapy-induced nausea and vomiting in glioblastoma patients treated with temozolomide: a phase II study. Support Care Cancer. 2011;19:697-701. 10.1007/s00520-010-0893-y 20467757

[20] National Comprehensive Cancer Network (NCCN). Antiemesis. NCCN Clinical Practice Guidelines in Oncology. Version 2.2023. Available at: https://www.nccn.org

[21] Zhang Y , Yang Y, Zhang Z, et al. Neurokinin-1 receptor antagonist-based triple regimens in preventing chemotherapy-induced nausea and vomiting: a network meta-analysis. J Natl Cancer Inst. 2017;109: djw217. 10.1093/jnci/djw217 27795228

[22] Mori-Vogt S , Blazer M. Palonosetron for the prevention of chemotherapy-induced nausea and vomiting. Expert Rev Anticancer Ther. 2013;13:919-936. 10.1093/jnci/djw217 23984894

[23] Oura K , Morishita A, Manabe T, et al. Efficacy of olanzapine as an antiemetic drug for transcatheter arterial chemoembolization for hepatocellular carcinoma. Sci Rep. 2025;2415:18095. 10.1038/s41598-025-01632-9 40413249 PMC12103546

[24] Hayashi T , Shimokawa M, Matsuo K, et al. Efficacy of 3-day versus 5-day aprepitant regimens for long-delayed chemotherapy-induced nausea and vomiting in patients receiving cisplatin-based chemotherapy. Expert Opin Pharmacother. 2023;24:2221-2226. 10.1080/14656566.2023.2288288 38009903

[25] Inui N , Toi Y, Yoneshima Y, et al. Pooled analysis of studies evaluating fosnetupitant and risk factors for cisplatin-induced nausea and vomiting during the extended overall phase. Adv Ther. 2023;40:4928-4944. 10.1007/s12325-023-02648-1 37715851 PMC10567891

[26] Caritis S , Zhao Y, Chen HJ, et al. Pharmacodynamics of transdermal granisetron in women with nausea and vomiting of pregnancy. Am J Obstet Gynecol. 2016;215:93.e1-4-93.e4. 10.1016/j.ajog.2016.01.163 26812081

