# Supplementary material for: Granisetron transdermal delivery system versus palonosetron in the prevention of long-delayed nausea and vomiting: a phase III randomized trial
Source: Oncologist. 2026 Jan 11;31(3):oyag007. doi: 10.1093/oncolo/oyag007 (PMC12923111; doi:10.1093/oncolo/oyag007)
Supplement: oyag007_Supplementary_Data [file oyag007_supplementary_data.docx]

Supplemental Table S1. Characteristics of the patients at baseline.

|  |  | Granisetron  (N = 150) | Palonosetron (N = 150) | |
| --- | --- | --- | --- | --- |
| Emetic Risk of Chemotherapy | High | 120 (80%) | 120 (80%) | |
|  | Moderate | 30 (20%) | 30 (20%) | |
| Age | Mean ± SD | 50.2 ± 10.4 | 49.3 ± 10.4 | |
| Height (cm) | Mean ± SD | 160.1 ± 5.1 | 160.6 ± 5.4 | |
| Weight (kg) | Mean ± SD | 60.2 ± 9.1 | 59.7 ± 7.9 | |
| Gender | Female | 147 (98%) | 145 (96.7%) | |
|  | Male | 3 (2%) | 5 (3.3%) | |
| ECOG | 0 | 37 (24.7%) | 26 (17.3%) | |
|  | 1 | 113 (75.3%) | 124 (82.7%) | |
| Cancer |  |  |  |  |
|  | Breast | 147 (98%) | 145 (96.7%) |  |
|  | Colon | 3 (2%) | 5 (3.3%) |  |
| Stage |  |  |  |  |
|  | I | 38 (25.3%) | 40 (26.7%) |  |
|  | II | 69 (46%) | 62 (41.3%) |  |
|  | III | 32 (21.3%) | 38 (25.3%) |  |
|  | IV | 11 (7.3%) | 10 (6.7%) |  |
| Chemotherapy regimen |  |  |  |  |
|  | AC | 74 (49.3%) | 70 (46.7%) |  |
|  | TAC | 4 (2.7%) | 3 (2.0%) |  |
|  | Carboplatin | 39 (26.0%) | 43 (28.7%) |  |
|  | Cisplatin-containg | 3 (2.0%) | 4 (2.7%) |  |
|  | TC | 27 (18%) | 25 (16.7%) |  |
|  | FOLFOX | 3 (2%) | 5 (3.3%) |  |
| Alcohol Consumption | Yes | 0 (0%) | 2 (1.3%) |  |
|  | No | 150 (100%) | 148 (98.7%) |  |
| Motion Sickness | Yes | 1 (0.7%) | 0 (0%) |  |
|  | No | 149 (99.3%) | 150 (100%) |  |
| Morning Sickness | Yes | 16 (10.7%) | 10 (6.7%) |  |
|  | No | 134 (89.3%) | 140 (93.3%) |  |
| Hypertension | Yes | 6 (4%) | 6 (4%) |  |
|  | No | 144 (96%) | 144 (96%) |  |
| Diabetes | Yes | 2 (1.3%) | 2 (1.3%) |  |
|  | No | 148 (98.7%) | 148 (98.7%) |  |
| Hepatitis | Yes | 6 (4%) | 5 (3.3%) |  |
|  | No | 144 (96%) | 145 (96.7%) |  |
| Previous Surgery | Yes | 89 (59.3%) | 79 (52.7%) |  |
|  | No | 61 (40.7%) | 71 (47.3%) |  |

^a^ t test ^b^ Chi-square test ^c^ Fisher’s exact test
